# Pain-Related Gene Solute Carrier Family 24 Member 3 Is a Prognostic Biomarker and Correlated with Immune Infiltrates in Cervical Squamous Cell Carcinoma and Endocervical Adenocarcinoma: A Study via Integrated Bioinformatics Analyses and Experimental Verification

**DOI:** 10.1155/2023/4164232

**Published:** 2023-02-07

**Authors:** Shuguang Zhou, Qinqin Jin, Hui Yao, Jie Ying, Lu Tian, Xiya Jiang, Yinting Yang, Xiaomin Jiang, Wei Gao, Weiyu Zhang, Yuting Zhu, Wujun Cao

**Affiliations:** ^1^Department of Gynecology, Anhui Province Maternity and Child Healthcare Hospital, Hefei, Anhui 230001, China; ^2^Department of Gynecology, Anhui Medical University Affiliated Maternity and Child Healthcare Hospital, Hefei, Anhui 230001, China; ^3^Department of Gynecology, Linquan Maternity and Child Healthcare Hospital, Fuyang, Anhui 236400, China; ^4^Department of Clinical Laboratory, Anhui Province Maternity and Child Healthcare Hospital, Hefei, Anhui 230001, China

## Abstract

The aim of this study was to explore cervical carcinoma and screen a suitable gene as the biomarker used for prognosis evaluation as well as pain therapy. Low expression levels of solute carrier family 24 member 3 (SLC24A3) was involved in the appearance and development of numerous malignancies. Nevertheless, the prognostic value of SLC24A3 expression with cervical squamous cell carcinoma and endocervical adenocarcinoma (CESC) patients remains uncertain. During the present study, SLC24A3 expression in CESC was retrieved from TCGA, GEO, and MSigDB databases. Based on TCGA and GEO profiles, we performed survival and difference analyses about SLC24A3 both in two GEO (GSE44001 and GSE63514) and TCGA-CESC cohorts (all *p* < 0.05), indicating that SLC24A3 was low expressed in tumors and associated with higher overall survival in CESC patients. Additionally, we programmed a series of analyses, including genomic profiling, enrichment analysis, immune infiltration analysis, and therapy-related analysis to identify the mechanism of the SLC24A3 in the process of cancer in CESC. Meanwhile, qRT-PCR was used to validate that the expression of SLC24A3 mRNA in Hela and SiHa cell lines was significantly lower than in PANC-1 and HUCEC cell lines. Our finding elucidated that the SLC24A3, a sodium-calcium regulator of cells, is an indispensable factor which can significantly influence the prognosis of patients with CESC and could provide novel clinical evidence to serve as a potential biological indicator for future diagnosis and pain therapy.

## 1. Introduction

Cervical carcinoma is the principal cause of mortality in female, specifically in some developing countries, and they are the second most universal cancer type in gynecology [1–5]. According to statistics, over half a million women are diagnosed with cervical carcinoma and the disease results in more than 300,000 deaths worldwide each year [6, 7]. Despite the increase in the incidence of cervical adenocarcinoma, cervical squamous cell carcinoma and endocervical adenocarcinoma (CESC) is still the most common type of cervical carcinoma. In relatively impoverished countries which lack formal screening programs and effective early diagnosis, CESC incidence and mortality are extremely high [8]. Recently, despite various studies have suggested that abnormally expressed tumor markers may be related to cancer occurrence and development tremendous endeavors to explore novel biomarkers, CESC continues to be a severe health problem among females. Hence, it is a matter of urgency to search novel prognostic biomarkers for female with CESC in order to improve the prognosis and reduce the mortality rate in CESC and to develop effective treatment indications for patients.

Cancer pain, simultaneously, could be the most common and unbearable symptoms in cancer patients, which has been shown to be associated with depression, anxiety, and reduced quality of life in cancer survivors of varied diagnoses [9]. Currently, the occurrence of cancer has been confirmed to be related to oncogenes and accompanied by pain in the body [10, 11]. Pain can be a common and uncontrollable symptom for tumor patients. It seriously affects the physical and psychological functions of patients [12, 13]. Although effective pain relief can be achieved in up to 90% of patients with cancer, numerous studies have shown that pain remains inadequately controlled in many patients [14, 15]. Thus, the study of pain-related genes in cancer patients can provide a basis for the development of targeted drugs for tumor therapy, which is of great significance to decrease pain in cancer patients and enhance their quality of life. CESC pain continues to be a major clinical challenge. Despite decades of thorough study and continuous efforts, the underlined cellular and molecular mechanisms remain elusive and the clinical approaches for treating CESC and cancer-related pain are limited. Therefore, this study attempts to explore new biomarkers that not only have a significant function in the prognosis and treatment of CESC patients but also have the potential to effectively control cancer pain.

## 2. Materials and Methods

### 2.1. Data Collection and Description

The Cancer Genome Atlas (TCGA) database (http://cancergenome.nih.gov/abouttcga/policies/publicationguidelines) was applied to download gene expression quantification data and clinical information of female with CESC. Nevertheless, because the TCGA database lacks normal tissue data matched with cervical cancer, the total number of samples is only three. Hence, the Genotype-Tissue Expression (GTEx) Portal (http://xena.ucsc.edu/) was applied to obtain the expression values of the normal cervix tissue. The Gene Expression Omnibus (GEO) database (https://www.ncbi.nlm.nih.gov/geo) was applied to download gene expression and clinical data of patients in GSE44001 and GSE63514 cohorts. The Molecular Signatures Database (*MSigDB*) (http://software.broadinstitute.org/gsea/msigdb/index.jsp) was used to extract pain-related genes. Since the data were downloaded from public databases, there is no need to get approval from ethics committee.

### 2.2. Identification of SLC24A3-Related Genes

RNA-seq data information of 306 tumor cases and 13 normal cases was obtained from the TCGA and GTEx official website. Differential expression genes (DEGs) were screened via “edgeR” R package by comparing SLC24A3 expression of the tumor and normal cases. We chose the *p* value < 0.05 and |log FoldChange| (|logFC|) > 1 as the cut-off criteria. We calculated the Spearman coefficients of the DEGs and key pain candidate genes, while defining DEGs with *p* value < 0.05 as key pain candidate genes.

### 2.3. Differential Methylation Analysis

306 tumor and 13 normal cases were chosen to perform differential methylation analysis. Probes containing single-nucleotide polymorphism (SNPs), probes with over 10% missing values, and probes in chromosome X were excluded from the analysis. And the “imputeTS” R package was applied to impute the missing values of the selected central pattern generators (CPGs). Differentially methylated CpGs (DMCs) were determined via the Wilcoxon rank-sum test, and the *p* values were adjusted via the false discovery rate (FDR) approach. If the mean methylation difference was >0.2 with an FDR of 5%, DMCs were reported.

### 2.4. Inference of Infiltrating Cells in CESC

The xCell algorithm, an R package (version 1.1.0), was applied to quantify the abundance of tumor stromal cells with CESC patients, which evaluates the integrated levels of 64 stromal cell types. xCell, a gene signature-based approach, combines the advantages of gene set enrichment with deconvolution methods, which well fits with RNA-seq and microarray data. And the gene expression data were prepared according to the xCell instructions, run R package, and undertaken using the xCell signature (*N* = 64) with 1000 permutations.

### 2.5. Drug Sensitivity Analysis

The Genomics of Drug Sensitivity in Cancer (GDSC) [16] database (https://www.cancerrxgene.org/) was used to download transcription data for almost 1000 cancer cell lines, drug response measurements as area under the curve (AUC) for antitumor drugs in cancer cell lines, and targets/pathways of drugs. The Spearman correlation analysis was performed to calculate the association between drug sensitivity and risk score. Then, we regarded |Rs| > 0.2 and applied Benjamini and Hochberg to adjust FDR and regarded an FDR < 0.05 as significant correlation. “pRRophetic” R package [17] was used to calculate the half-maximum inhibitory concentration (IC_50_) for comparing the drug sensitivity.

### 2.6. TMB and IPS Analysis

The “mafools” R package [18] was applied to calculate tumor mutation burden (TMB) from datasets of somatic mutations. The immunophenoscore (IPS) from The Cancer Immunome Atlas is a machine learning-based immune response score. The higher IPS score is associated with better immune response [19].

### 2.7. Cell Lines

Hela and SiHa are human cervical cancer cell lines which were used as the test groups. Among them, Hela was a cell line of cervical adenocarcinoma and SiHa was a cell line of cervical squamous cell carcinoma. We utilized PANC-1, a cell line of pancreatic cancer, as a positive control for SLC24A3 expression. Meanwhile, we utilized HUCEC, a cell line of normal cervical, as a negative control for SLC24A3 expression. All of them were obtained from the American Type Culture Collection (ATCC, Manassas, VA, United States). We used DMEM contained with 10% FBS (Gibco, Grand Island, NY) to incubate Hela and SiHa cells. Then, we used RPMI 1640 supplementing 10% FBS (Gibco, Grand Island, NY) to incubate PANC-1 and HUCEC cells. The cells are cultured in an incubator at 37°C with 5% CO2.

### 2.8. Quantitative Real-Time Polymerase Chain Reaction (qRT-PCR)

We used TRIzol reagent (Invitrogen, Carlsbad, CA) to collect and lyse cells. Then, M-MLV reverse transcriptase (Promega, Madison, WA) was utilized to obtain cDNA. Utilizing a LightCycler 480 II RT-PCR System (Roche, Basel, Switzerland), qRT-PCR was carried out as follows: 95°C for half a minute and then 40 cycles of 95°C for 5 seconds and 60°C for half a minute. In our study, glyceraldehyde-3-phosphate dehydrogenase (GAPDH) gene was utilized as an internal control; sequences of primers amplified in this study were contained in Table S3.

### 2.9. Data Processing and Analysis

In this study, R programming language was the major tool for processing data. The DEGs between CESC samples and healthy controls were acquired via the “EdgeR” R package, whereas |logFC| > 2 was screened as differentially expressed pain-related genes. The box plot and the forest plot were conducted by the “ggpubr” and “forestplot” R packages, respectively. Gene Ontology (GO) and Kyoto Encyclopedia of Genes and Genomes (KEGG) analyses were done via the “enrichplot” R package. Survival curves and survival analysis were conducted by the “survminer” as well as “survival” R packages. “Clusterprofiler” R package was applied for quantification of gene set activity on the gene set enrichment analysis (GSEA). The version of R bioconductor utilized in this study was R 4.1.2. The qualitative data were analyzed by *t*-test via SPSS 26.0.

## 3. Results

### 3.1. Screening for Pain-Related Genes in Cervical Cancer

**Figure 1:**
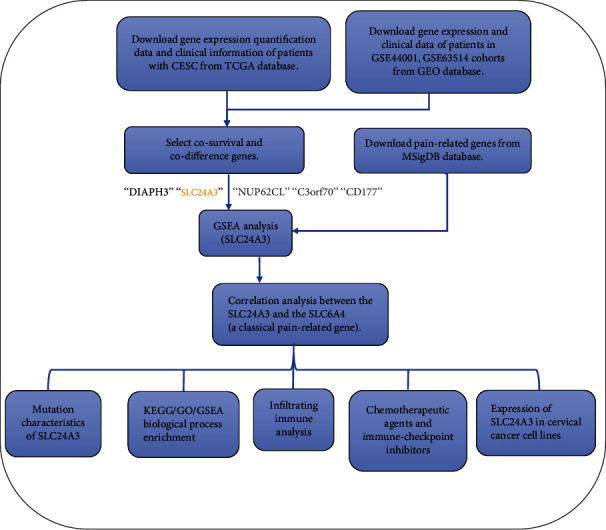
The flow chart of this study.

The flow chart of this study is presented in Figure 1. We performed difference analysis and survival analysis according to the CESC cohort from the TCGA database (combine with GTEx), including 306 tumor cases with survival data, RNA-seq data, and clinicopathological data. Then, we processed and analyzed 2 cervical squamous cell carcinoma datasets (GSE44001 and GSE63514) from the GEO platform, identifying differential expression genes and survival data between cancer and normal tissues, respectively. By integrating survival data from the TCGA and GEO cohorts, a total of 5 (DIAPH3, SLC24A3, NUP62CL, C3orf70, and CD177) DEGs (*p* < 0.05) were screened to perform the further GSEA analysis.

**Figure 2:**
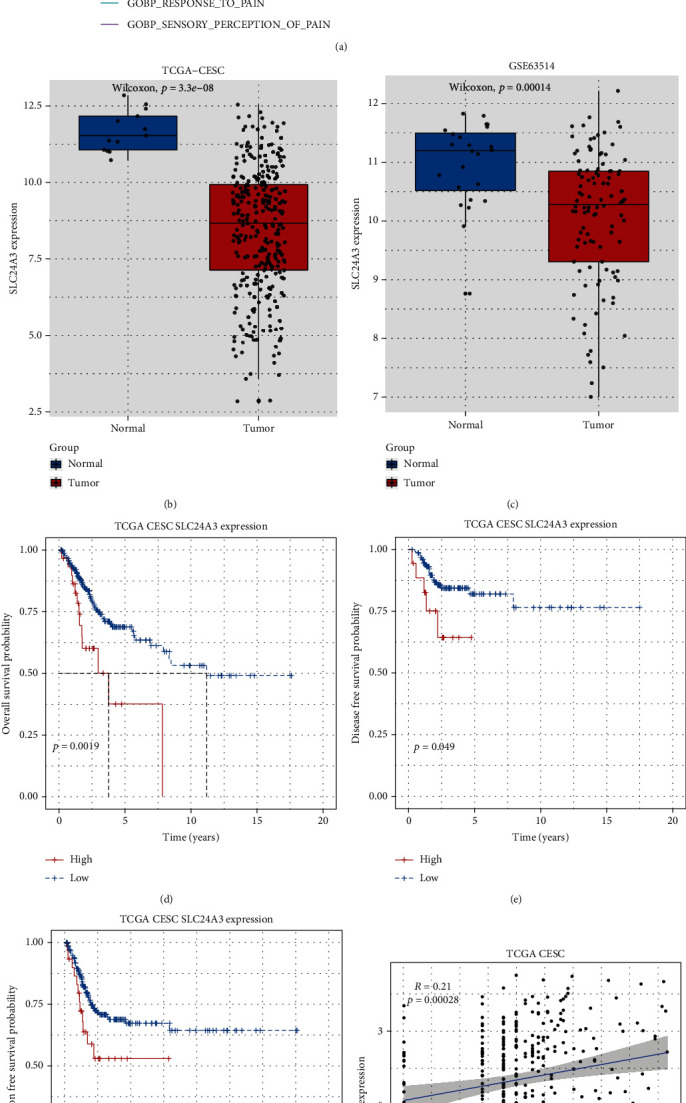
Pain-related gene selection: (a) GSEA for SLC24A3. (b, c) Expression levels of SLC24A3 between tumor and normal cases with CESC patients both in TCGA-CESC and GSE63514 cohorts. (d–f) Overall survival, disease-free survival, and progression survival comparisons between high and low SLC24A3 groups in TCGA database. (g, h) Correlation analysis between SLC24A3 and SLC6A4 in TCGA and GSE63514 cohorts.

There were 117 pain-related genes acquired from *MSigDB* (Table S1). According to the results of GSEA analysis, there was only one gene enriched in pain-related pathway, which is SLC24A3 (Figure 2(a)). As illustrated in box plot, SLC24A3 expression in tumor groups was all considerably lower than nontumor groups in TCGA-CESC and GSE63514 cohorts (all *p* < 0.05; Figures 2(b) and 2(c)). The results demonstrated that downregulation of SLC24A3 in tumor tissues was greatly associated with better overall survival (log rank *p* = 0.0019; Figure 2(d)), disease-free survival (log rank *p* = 0.049; Figure 2(e)), and progression-free survival (log rank *p* = 0.049; Figure 2(f)) among patients with CESC.

Additionally, correlation analysis indicated that the SLC24A3-expressed levels were positively related to the SLC6A4-expressed level both in TCGA and GSE63514 cohorts (all *p* < 0.05; Figures 2(g) and 2(h)). Among them, SLC6A4 is a classical pain-related gene. In the same vein, we performed expression levels and survival analyses with SLC6A4 (Figure S1 A-E), which outlined similar results with SLC24A3. Finally, we applied the Human Protein Atlas (HPA) database to validate the SLC24A3 expression in pathological and healthy cervical tissues, finding that there was an obviously differential expression of SLC24A3 in pathological cervical tissues and healthy cervical tissues (Figure S1 F-H).

### 3.2. Mutation Characteristics of SLC24A3 Genome

**Figure 3:**
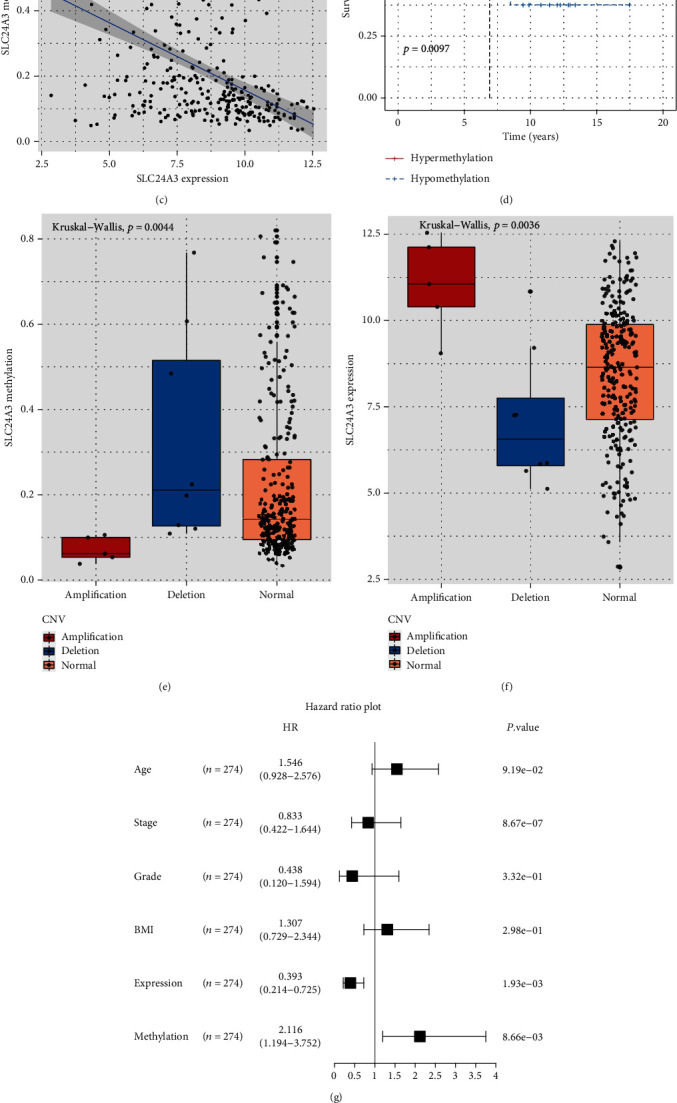
Landscape of genetic and expression variations of SLC24A3 in CESC. (a) The mutation frequency of SLC24A3 in CESC. (b) The Kaplan-Meier survival curve estimated in accordance with amplification, deletion, and normal SLC24A3-mutated tumor of CNV. (c) The association between copy number of variation derived from methylation and expression of SLC24A3. (d) The overall survival curve of methylation status of SLC24A3. (e) Box plot showed the relation between methylation status of SLC24A3 and CNV amplification (red), deletion (blue), and non-CNV (orange) in the TCGA-CESC cohort. (f) Box plot showed the relation between SLC24A3 expression and CNV amplification (red), deletion (blue), and non-CNV (orange) in the TCGA-CESC cohort. (g, h) The forest plots for univariate Cox regression analysis and multivariate Cox regression analysis in TCGA-CESC cohort.

Mutation panorama of SLC24A3 showed that somatic mutation rate was 2.08% and accounted for 5 sites in SLC24A3 (Figure 3(a) and Figure S2 A). However, SLC24A3 with copy number variations (CNV) of these amplifications had lower survival probability than those CNV deletion and without CNV mutations (*p* < 0.0001; Figure 3(b)), suggesting that genetic alteration of CNV amplification mutation might play certain functional role in CESC. Then, we used R project to perform differential methylation analysis in both CESC and normal cervix tissues, finding that hypermethylated SLC24A3 was adversely associated with SLC24A3 expression (Figure 3(c)) and hypermethylated SLC24A3 considerably contributed to a well overall survival probability in CESC patients (*p* = 0.0097; Figure 3(d)). Box plot showed the significantly correlation between methylation status of SLC24A3 and CNV deletion (*p* = 0.0044; Figure 3(e)). Moreover, to ascertain how CNV impacted the expression of SLC24A3 among CESC patients, we compared the SLC24A3-expressed levels between CNV amplification mutations, deletion mutations, and normal samples and showed that the CNV amplification mutations was significantly increased in CESC (Figure 3(f)). By the same token, we performed analyses about survival and expression of SLC24A3 derived from single-nucleotide variant (SNV) mutations, and the results were similar as CNV (Figure S2 B-C). Clinical characteristics of TCGA CESC data between the CNV and SNV groups are shown in Table S2.

Then, we performed the univariate Cox regression analysis using age, grade, stage, TNM status, body mass index (BMI), methylation status, and SLC24A3-expressed levels as inputs; our analysis proved that methylation status and the expression of SLC24A3 were significantly associated with the survival outcomes (*p* < 0.05; Figure 3(g)). Subsequently, we also performed the multivariate Cox regression analysis using stage, methylation status, and SLC24A3-expressed levels as inputs; the results exposed that the SLC24A3-expressed level was a robust and independent predictor of enhanced prognosis (hazard ratio: 0.214-0.725, *p* = 1.93*e* − 03; Figure 3(h)).

### 3.3. KEGG/GO/GSEA Biological Process Enrichment

**Figure 4:**
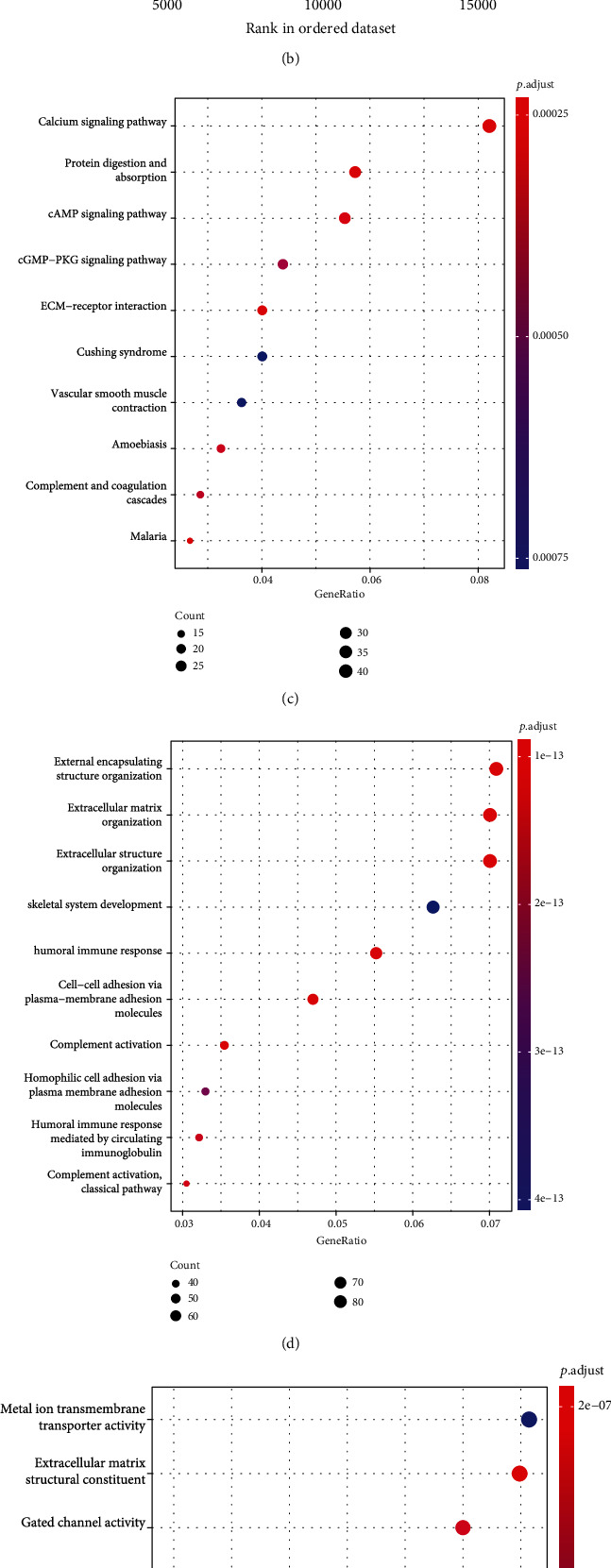
Functional enrichment of SLC24A3-related genes. (a) Volcano plot of differential SLC24A3-related genes and |logFC| > 1 with *p* value < 0.05 were utilized as screening criteria for DEGs. (b) KEGG signal pathway enrichment analysis. (c) Biological process enrichment analysis, (d) cell component enrichment analysis, and (e) molecular function enrichment analysis. (f) Similarly, GSEA identified a set of genes associated with SLC24A3.

Volcano plot indicated differential SLC24A3-related genes. Notably, there were 1,771 DEGs overlapped among the three cohorts, which included 774 upregulated genes and 937 downregulated genes (|logFC| > 1 and *p* < 0.05; Figure 4(a)). The KEGG pathway enrichment of SLC24A3 interactive genes revealed that cysteine and methionine metabolism, glutathione metabolism, glyoxylate and dicarboxylate metabolism, purine metabolism, pyrimidine metabolism, and pyruvate metabolism were the significantly enriched pathways (Figure 4(b)). In addition, GO analysis results indicated that SLC24A3-related genes were mostly enriched in the calcium signaling pathway, ECM-receptor interaction, cAMP signaling pathway, protein digestion and absorption, etc. at biological process (BP) levels; extracellular matrix organization, external encapsulating structure organization, extracellular structure organization etc. at cellular components (CC) levels; and gated channel activity, extracellular matrix structural constituent, glycosaminoglycan binding, antigen binding, etc. at molecular function (MF) levels (Figures 4(c)–4(e)). GSEA identified a set of genes associated with DNA repair, mitochondrion organization, NCRNA metabolic process, and reactome cell cycle mitotic (Figure 4(f)).

### 3.4. Associations of SLC24A3 with Tumor-Infiltrating Immune Cells and Immune-Related Gene Sets

**Figure 5:**
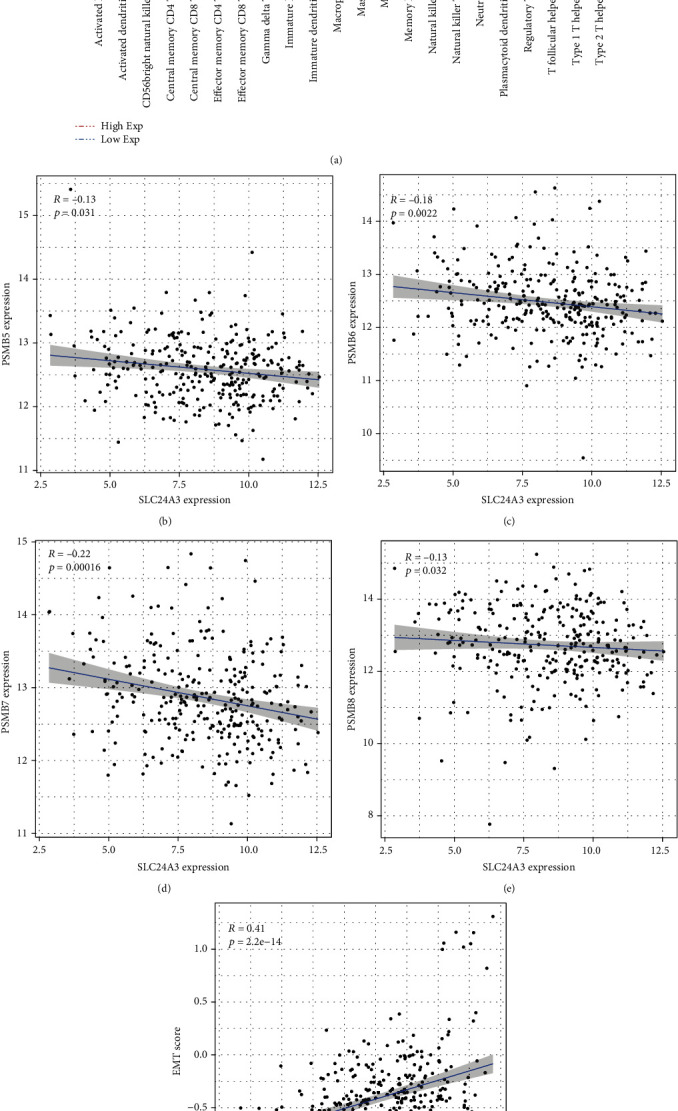
The correlation between SLC24A3 and immune infiltration (xCell). (a) Violin plot of association between the abundance of immune cells and SLC24A3 expression in CESC. (b–e) The association between SLC24A3 expression and antigen-presenting cell gene set. (f) The association between SLC24A3 expression and EMT score.

In our study, the comprehensive levels of 64 immunity and stromal cell types of 274 CESC samples were calculated. Among immune infiltration analysis of 64 immune cells, the xCell algorithm results elucidated that proportion of activated B cell (*p* = 1.44*e* − 03), activated dendritic cell (*p* = 1.72*e* − 02), CD56 bright natural killer cell (*p* = 5.62*e* − 03), central memory CD4 T cell (*p* = 4.5*e* − 05), central memory CD8 T cell (*p* = 1.12*e* − 06), effector memory CD4 T cell (*p* = 2.16*e* − 05), effector memory CD8 T cell (*p* = 1.33*e* − 02), gamma delta T cell (*p* = 4.73*e* − 02), immature B cell (*p* = 4.66*e* − 03), immature dendritic cell (*p* = 5.23*e* − 05), macrophage (*p* = 1.03*e* − 03), mast cell (*p* = 2.92*e* − 02), MDSC (*p* = 2.05*e* − 03), memory B cell (*p* = 3.37*e* − 02), natural killer cell (*p* = 2.88*e* − 04), natural killer T cell (*p* = 2.41*e* − 03), neutrophil (*p* = 1.33*e* − 02), plasmacytoid dendritic cell (*p* = 5.08*e* − 04), regulatory T cell (*p* = 1.27*e* − 03), T follicular helper cell (*p* = 4.25*e* − 06), type 1 T helper cell (*p* = 6.77*e* − 05), type 2 T helper cell (*p* = 4.58*e* − 02), a total of 22 immune cells, was enriched in SL24A3 high-expressed group (Figure 5(a)). This evidence suggested a significant correlation between SLC24A3 and tumor-immune infiltration. Additionally, the results of correlation analysis between SLC24A3-expressed levels and antigen-presenting cells gene sets indicated that the expression levels of PSMB5, PSMB6, PSMB7, and PSMB8 were increased in SLC24A3 low-expressed group (Figures 5(b) and 5(e)). Moreover, the expression level of SLC24A3 was positively correlated to epithelial-mesenchymal transition (EMT), which implied that SLC24A3 was a contributor to EMT regulation in CESC (Figure 5(f)).

### 3.5. The Prediction of SLC24A3 for Response to Chemotherapeutic Agents and Immune Checkpoint Inhibitors (ICIs)

**Figure 6:**
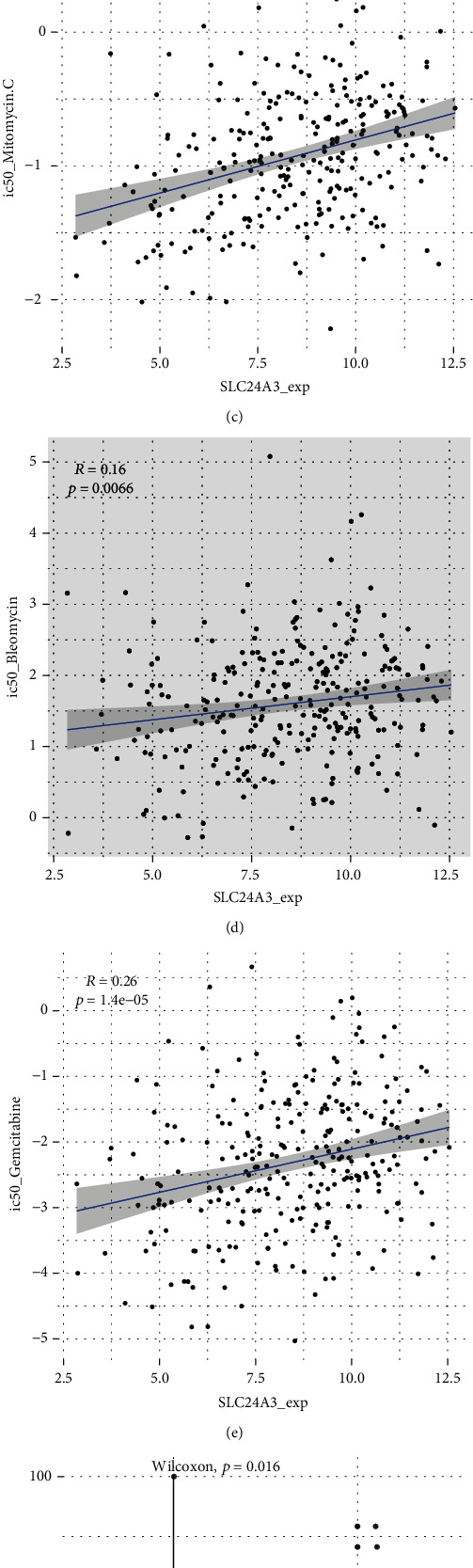
The prediction of SLC24A3 for response to chemotherapeutic agents and immune checkpoint inhibitors. (a) The association between risk score and drug sensitivity estimated by the Spearman analysis. (b) Signaling pathways targeted by drugs that are sensitive (blue) or resistant (red) to the risk score. (c–e) The correlation between SLC24A3 expression and drug sensitivity in CESC. (f, g) Box plots showed differences in TMB (f) and IPS (g) between the SLC24A3 mutation (red) and nonmutation (blue) groups in the TCGA-CESC cohort.

To fully comprehend the influence of the risk score on drug response, we estimated the relationship between the risk score and the response to drugs in tumor cell lines. So, we applied the Spearman correlation analysis to find 64 greatly associated pairs between risk score and drug sensitivity in GDSC database (Figure 6(a)). 42 pairs of which exhibited that drug sensitivity associated with the risk score, which included CCT007093, imatinib, and DMOG. 22 pairs of drug showed resistance associated with the risk score, including mitomycin C and tipifarnib. Furthermore, analyzing the signaling pathways of the genes targeted by these drugs, we identified that drugs whose sensitivity was related to the high-risk score were mainly targeting PI3K/MTOR, JNK and p38, and DNA replication signaling pathways. Conversely, the drug whose sensitivity was related to the low-risk score was mostly targeting metabolism, cell cycle, and WNT signaling pathway (Figure 6(b)).

Thereafter, we performed a study to investigate the effects of SLC24A3 with chemotherapies and immunotherapies. We utilized “pRRophetic” R package to predict therapeutic effect of 168 drugs. Among them, only 3 IC_50_ of drugs were significantly correlated with SLC24A3 expression, including mitomycin C, bleomycin, and gemcitabine (Figures 6(c)–6(e)). That meant worse expression of SLC24A3 could enhance the effect of chemotherapy drug treatment. We applied the IPS to predict ICI treatment effectiveness among CESC patients categorized as mutation and nonmutation group according to the mutation of SLC24A3 (Figure 6(g)). The nonmutation group showed superior IPS than the mutation group, which represented superior immunogenicity, thus implying a greater response to ICIs. But CESC patients with mutations of SLC24A3 had higher TMB than those without mutations (Figure 6(f)). Together, the SLC24A3 showed a considerable therapeutic value in predicting ICI treatment effect and outstanding potential drug targets.

### 3.6. Expression of SLC24A3 in Cervical Cancer Cell Lines

**Figure 7:**
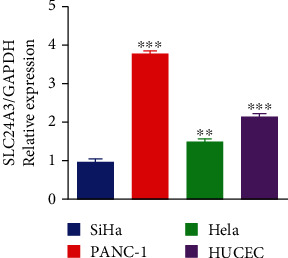
Expression of SLC24A3 mRNA in cervical cancer cell lines. The expression levels of SLC24A3 mRNA were significantly decreased in Hela and SiHa cell lines compared with the PANC-1 and HUCEC groups. Error bars represent ±SD. ^∗^*p* < 0.05, ^∗∗^*p* < 0.01, and ^∗∗∗^*p* < 0.001. PANC-1 was utilized as a positive control. HUCEC was utilized as a negative control.

qRT-PCR revealed that the levels of SLC24A3 mRNA were obviously downregulated in Hela and SiHa cell lines compared with PANC-1 and HUCEC cell lines (Figure 7, *p* < 0.01). That consisted with our bioinformatics analysis and further validated that the SLC24A3 was lower expressed among patients with CESC.

## 4. Discussion

Cervical carcinoma is the main reason of death among female in developing countries and areas, and there are over half a million new cases and 300,000 fatalities per year [20]. It accounts for about 10-15% of all woman cancer-related deaths globally and is the second most cause of cancer fatalities in woman, after breast cancer [21, 22]. Due to the lack of efficient early noninvasive screening, 80% of people have progressed to the invasive cancer stage of cervical cancer when were diagnosed. Meanwhile, surgery is vital for partly cancer; however, 10-80% of people suffer pain after diverse kinds of surgery. As far as we know, cancer pain refers to pain directly caused by tumor and the most turbulent influence on the quality of life of patients. Previous studies have reported that tumor invades or compresses nerve root, nerve trunk, nerve plexus, or nerve, invades brain and spinal cord, invades periosteum or bone; invades substantial organs and cavernous organs, invades or blocks vascular system, and causes local necrosis, ulceration, inflammation, etc., which can result in severe pain in all the above cases [23, 24]. Of cause, the conventional treatment of cancer pain is on basis of the WHO recommended “three-step therapy”; unfortunately, it lacks effectiveness at the terminal stage [25–27]. Although an increasing number of advances are being made in cancer treatment and diagnosis, the results of treatment are not very satisfactory as we expected. At the same time, promising insights into biomarker are now arising, and have the potential to effectively control cancer pain. Therefore, in this essay, we explore a new biomarker SLC24A3, which has functions not only in prognostic as well as treatment but also in relief cancer pain of patients with CESC.

Firstly, we downloaded CESC cohort from TCGA and GEO databases. In this study, by integrating the two GEO and TCGA-CESC cohorts, we found five difference expression genes (DIAPH3, SLC24A3, NUP62CL, C3orf70, and CD177). Then, we retrieved pain-related gene from MSigDB database. The GSEA analysis revealed that only one gene was enriched in the pain pathway. Thus, we successfully screened out the gene SLC24A3. This study manifested that SLC24A3 had a considerable difference of expression in tumor and normal tissues of CESC, and we also proved that low-expressed SLC24A3 has well survival rates in patients with CESC. Intriguingly, we found that SLC24A3 has significantly positive correlation with a classical pain gene—SLC6A4. Previous studies have indicated that the human 5-HT transporter (5-HTT) gene (SLC6A4) features some polymorphisms in its promoter region (5-HTTLPR) that impact the 5-HTT expression [28, 29]. Increasing evidence exhibited that serotonin (5-HT) is hugely correlated with pain regulation [30]. Given that SLC6A4 was highly associated with pain regulation and SLC24A3 has positively correlation with SLC6A4, we hypothesized that SLC24A3 was associated with cancer pain in CESC.

To verify the genetic alterations in SLC24A3 in CESC, we drew a panorama gene mutation showing the location and number of somatic mutations in SLC24A3. Copy number variations (CNV) are a usual form of genetic instability, which could directly alter the expression level of particular genes, which plays essential roles in susceptibility to disease [31, 32]. In addition, somatic (cancer-only) single-nucleotide variants (SNVs) as another form of mutation are the simplest type of mutation; however, their identification in DNA sequencing data is interfaced by tumor heterogeneity and sequencing, germline polymorphisms, and analysis errors [33, 34]. No matter mutations derived from CNV or SNV, all results demonstrated that the amplification mutations are positively relative with the expression level of SLC24A3. Likewise, the Kaplan-Meier survival curve revealed that SLC24A3 presenting with amplification mutations was associated with worse survival rate. Subsequently, we investigated the effect of genomic features on methylation and found that the SLC24A3 low-expressed group was highly methylated. According to the findings of univariate and multivariate Cox regression analyses, the SLC24A3-expressed level was an independent protective factor for CESC.

To further understand the functional role of the SLC24A3, GO, KEGG, and GSEA were performed, and the results indicated that SLC24A3 was greatly enriched in biological processes such as DNA repair, mitochondrion organization, NCRNA metabolic process, and reactome cell cycle mitotic.

Immune infiltration of tumors is intimately related to clinical consequences in CESC. Tumor-infiltrating immune cells (TIIC) form an ecosystem in the tumor microenvironment to modulate cancer development and have exhibited underlying prognostic value [35]. Tumor cells interact with their microenvironment and are affected by signals coming from stromal, inflammatory, immune, and endothelial cells. Certainly, tumors are usually infiltrated by diverse kinds of lymphocytes, mast cells, or macrophages [36]. Conventional wisdom is that lymphocytes could control cancer outcome, whereas the latter produce factors that promote tumor growth and maintain chronic inflammation [37]. To examine whether the expression of SLC24A3 could reflect the immune microenvironment, we performed immune infiltration of tumors analysis by using xCell algorithm. And the result implied that there were 22 immune cells enriched in the group with SLC24A3 highly expressed. In addition, correlation analysis results elucidated that SLC24A3 was a certain contributor to PSMB5, PSMB6, PSMB7, PSMB8, and EMT regulation in CESC. These changes were involved in angiogenesis in the tumor microenvironment, strengthening the invasion and metastasis of CESC cells [38, 39].

Drug sensitivity analysis was performed to validate whether risk score could predict patients' sensitivity to chemotherapy. The results of drug analysis indicated that the therapeutic effect of mitomycin C, bleomycin, and gemcitabine could be improved when the SLC24A3 was low expressed. TMB and IPS were applied to evaluate the potential response of immune checkpoint blockade (ICB) therapies. The immunotherapy results revealed that the higher the TMB and IPS, the better the immunotherapy effect. In summary, these findings may be beneficial to clinical decision-making.

The results of qRT-PCR demonstrated that the SLC24A3 mRNA was lower expressed in Hela and SiHa cell lines compared with PANC-1 (a positive control) and HUCEC (a negative control) cell lines. It was noteworthy that SLC24A3 mRNA was significantly higher expressed in PANC-1 and serves as human pancreatic cancer cell lines, compared with HUCEC cell lines. This suggested that SLC24A3 was differentially expressed in different cancer type compared with normal control tissues. As far as we know, however, no previous research has investigated whether SLC24A3 is involved in occurrence and development of pancreatic cancer. Hence, we were unable to understand how SLC24A3 exerted its function in pancreatic cancer during occurrence and development. Nonetheless, that did not influence our conclusion that the experimental results of qRT-PCR consisted with the above bioinformatics analysis obviously.

Further, in the existing studies, scientists have revealed that Nckx3 (gene SLC24A3), one of the six isoforms of Na+/K+/Ca2+ exchanger family, is able to operate either in forward or in reverse mode relying on both ion gradients and membrane potential [40–42]. Besides, transcripts of Nckx3 have been discovered in diverse other excitable body tissues with abundant smooth muscle, including the lung, intestine, aorta, and uterus [43]. Naureen et al. have reported in early research that SLC24A3 was associated with multisite chronic pain [44]. Meanwhile, SLC24A3 has been associated with some cancers. Previous studies have illustrated that SLC24A3 was significantly differentially expressed in colon cancer, ovarian cancer, and cervical cancer compared with normal control tissues [45, 46]. Gormley et al. demonstrated that SLC24A3 is a pain-related gene [47]; unfortunately, there is limited literature on the direct connection between SLC24A3 and cancer pain, and its involvement in the mechanism of initiation and progression of cancer pain is yet unclear. The existing research about SLC24A3 mainly focuses on its own chemical mechanism, although we lack detailed clinical and experimental data to accurately estimate whether SLC24A3 has the potential to alleviate the biomarker of CESC pain. Based on the available research results, we have reason to believe that upregulating the expression of SLC24A3 will effectively alleviate the cancer pain in CESC. In other words, cancer pain is triggered precisely because SLC24A3 is low expressed in CESC, which may be due to the alteration of inflammation-related immune mechanisms. Just as we found, the deletion of Nckx3 exacerbated experimental DSS-induced mouse colitis through the p53/NF-*κ*B pathway [48]. At the same time, based on the research we did to explore the connection between SLC24A3 and tumor-infiltrating immune cells and immune-related gene sets, we found that SLC24A3 can affect the tumor-immune microenvironment, which indicates that SLC24A3 may control the body's pain response by immunomodulation. Thus, taking all our results into consideration, the SLC24A3, as a pain-related gene, may indeed be a well biological indicator for CESC patients. This introduces a promising direction for our subsequent research, for the propose of further elucidating and understanding the mechanism of SLC24A3 in relieving cancer pain. Verifying whether SLC24A3 is appropriate as a biological indicator in early diagnosis, evaluation of prognosis and relief cancer pain of CESC patients is extremely essential.

In conclusion, firstly, we retrieved five pain-related genes from TCGA, GEO, and *MSigDB* databases. Then, SLC24A3 was obtained, enriched in the pain signaling pathway by GSEA. Subsequently, genomic profiling, enrichment analysis, immune infiltration, and chemotherapy and immunotherapy analysis were performed. All those gene bioinformatics suggested that SLC24A3 could be a possible molecular targeting mechanism for the prevention and pain treatment of CESC, which needs to be tested in further clinical trials.

Admittedly, some limitations indeed exist in this study. First, gene expression profile utilized in present study was from different platforms; this discrepancy may produce bias in the analysis process. Moreover, experiments to illustrate the underlying function and mechanism of SLC24A3 in the therapy of cervical cancer pain were not performed yet. Last but not least, we need to conduct in vitro model and experimental studies to validate the assumption we proposed according to the functions of the SLC24A3, along with the predicted drugs. Therefore, the function of SLC24A3 of CESC is encouraging enough to warrant advanced exploration.

## Data Availability

*Data Collection and Description*. The Cancer Genome Atlas (TCGA) database (http://xena.ucsc.edu/) was applied to download gene expression quantification data and clinical information of female with CESC. Nevertheless, because the TCGA database lacks normal tissue data matched with cervical cancer, the total number of samples is only three. Hence, the Genotype-Tissue Expression (GTEx) Portal was applied to obtain the expression values of the normal cervix tissue. The Gene Expression Omnibus (GEO) database (https://www.ncbi.nlm.nih.gov/geo) was applied to download gene expression and clinical data of patients in GSE44001 and GSE63514 cohorts. The Molecular Signatures Database (MSigDB) (http://software.broadinstitute.org/gsea/msigdb/index.jsp) was used to extract pain-related genes. Since the data were downloaded from public databases, there is no need to get approval from ethics committee.

## Supplementary Materials

Supplementary Materials Figure S1: correlation analysis of SLC6A4 in GSE63514 and TCGA-CESC cohorts. (A, B) Expression levels of SLC6A4 between tumor and normal cases both in TCGA-CESC and GSE63514 cohorts. (C–E) Overall survival, disease-free survival, and progression survival comparisons between high and low SLC6A4 groups in TCGA-CESC database. (F–H) The SLC24A3 expression in pathological cervical tissues and healthy cervical tissues from the Human Protein Atlas database. Figure S2: analysis of SNV mutations of SLC24A3. (A) The alteration frequency of SLC24A3. (B) The Kaplan-Meier curve showed overall survival probability of patients with (red) or without (blue) SNV mutations of SLC24A3 in TCGA-CESC cohort. (C) Box plot showed the relation between SLC24A3 expression and SNV mutations in TCGA-CESC cohorts. Supplementary Table 1: 117 pain-related genes from *MSigDB*. Supplementary Table 2: clinical characteristics of TCGA CESC data between the CNV and SNV groups. Supplementary Table 3: primer sequence of SLC24A3 used in qRT-PCR.
