# Supplementary material for: Pain-Related Gene Solute Carrier Family 24 Member 3 Is a Prognostic Biomarker and Correlated with Immune Infiltrates in Cervical Squamous Cell Carcinoma and Endocervical Adenocarcinoma: A Study via Integrated Bioinformatics Analyses and Experimental Verification
Source: Comput Math Methods Med. 2023 Feb 7;2023:4164232. doi: 10.1155/2023/4164232 (PMC9928512; doi:10.1155/2023/4164232)
Supplement: Supplementary Materials — Figure S1: correlation analysis of SLC6A4 in GSE63514 and TCGA-CESC cohorts. (A, B) Expression levels of SLC6A4 between tumor and normal cases both in TCGA-CESC and GSE63514 cohorts. (C–E) Overall survival, disease-free survival, and progression survival comparisons between high and low SLC6A4 groups in TCGA-CESC database. (F–H) The SLC24A3 expression in pathological cervical tissues and healthy cervical tissues from the Human Protein Atlas database. Figure S2: analysis of SNV mutations of SLC24A3. (A) The alteration frequency of SLC24A3. (B) The Kaplan-Meier curve showed overall survival probability of patients with (red) or without (blue) SNV mutations of SLC24A3 in TCGA-CESC cohort. (C) Box plot showed the relation between SLC24A3 expression and SNV mutations in TCGA-CESC cohorts. Supplementary Table 1: 117 pain-related genes from MSigDB. Supplementary Table 2: clinical characteristics of TCGA CESC data between the CNV and SNV groups. Supplementary Table 3: primer sequence of SLC24A3 used in qRT-PCR. [file 4164232.f1.zip › Figure S2(A-C).pdf]

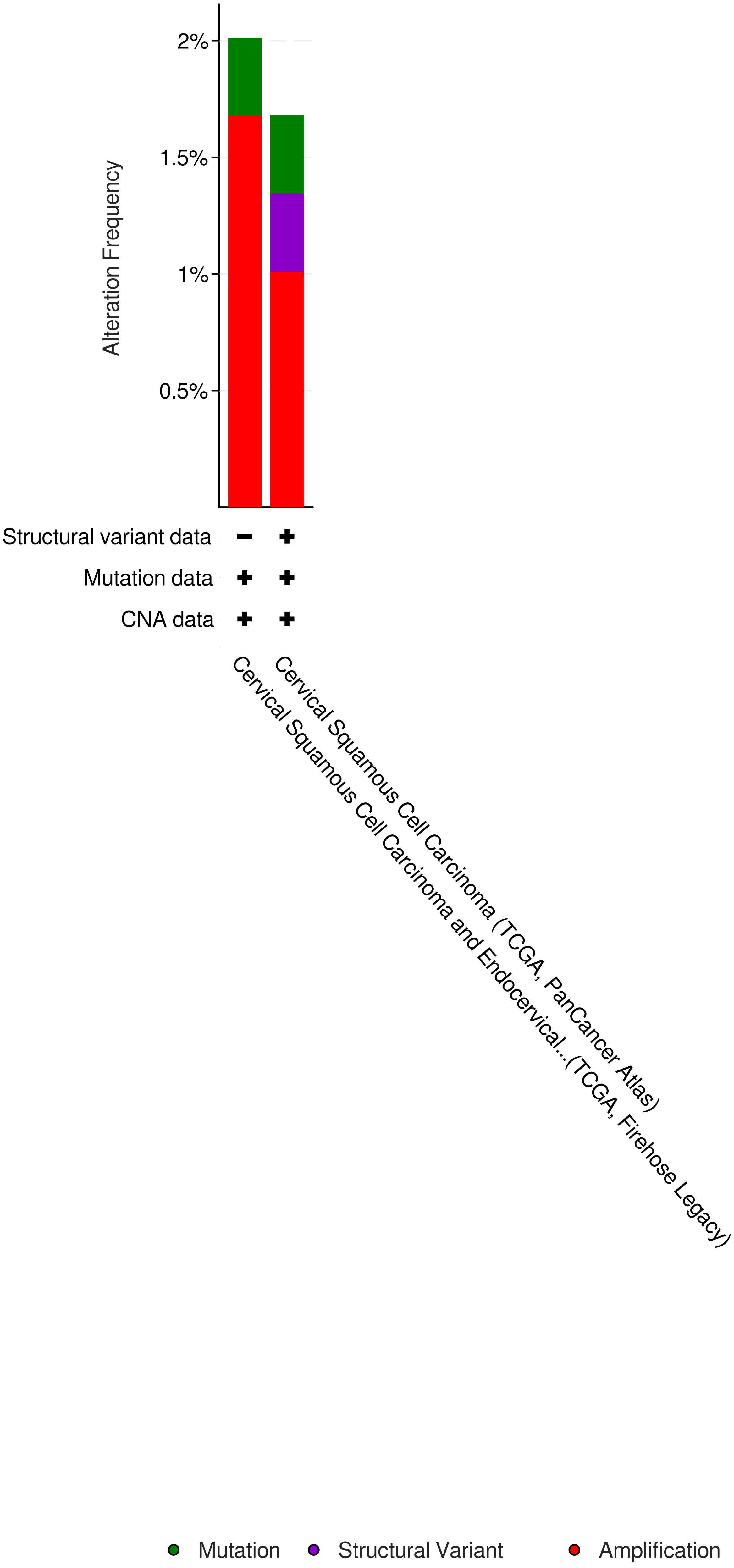

Alteration Frequency
2%
1.5%
1%
0.5%
Structural variant data
Mutation data
CNA data
Cervical Squamous Cell Carcinoma and Endocervical...(TCGA, Firehose Legacy)
Cervical Squamous Cell Carcinoma (TCGA, PanCancer Atlas)
Mutation
Structural Variant
Amplification

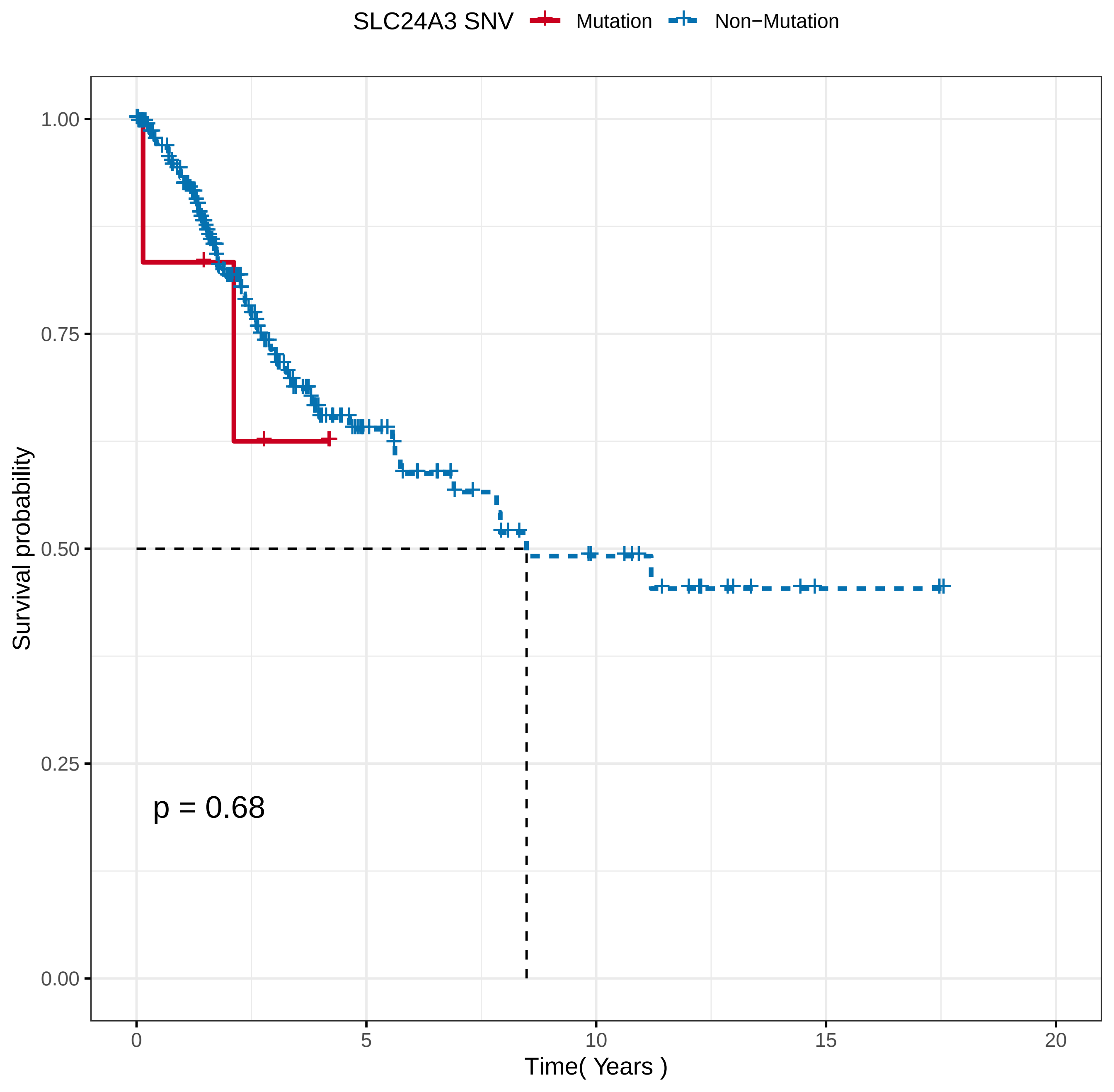

SLC24A3 SNV
Mutation
Non−Mutation
Survival probability
1.00
0.75
0.50
0.25
0.00
p = 0.68
0
5
10
15
20
Time(Years)

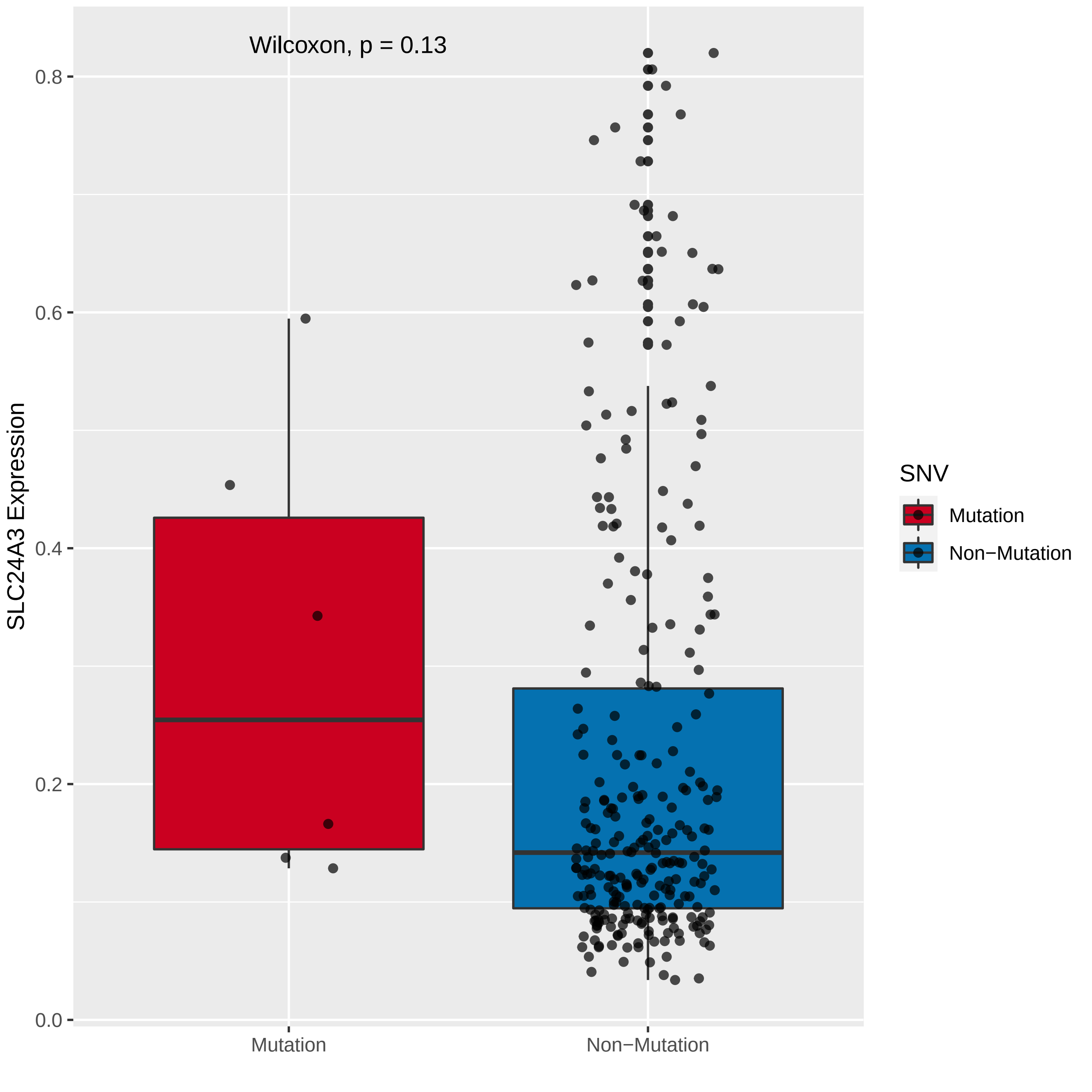

Wilcoxon, p = 0.13
SLC24A3 Expression
0.8
0.6
0.4
0.2
0.0
Mutation
Non−Mutation
SNV
Mutation
Non−Mutation

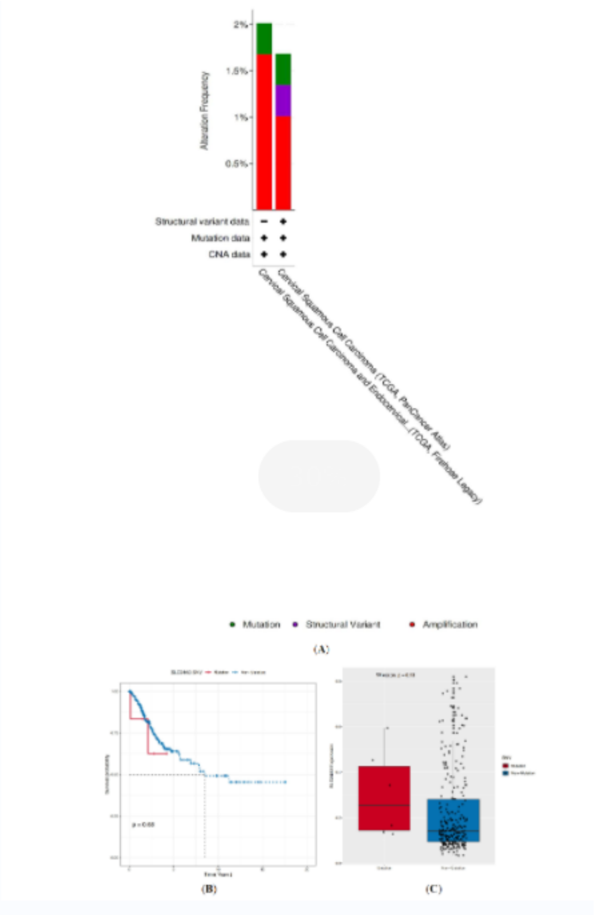

Supplementary 2. Figure S2. Analysis of SNV mutations of SLC24A3. (A) The alteration frequency of SLC24A3. (B) Kaplan-Meier curve showed overall survival probability of patients with (red) or without (blue) SNV mutations of SLC24A3 in TCGA-CESC cohort. (C) Box plot showed the relation between SLC24A3 expression and SNV mutations in TCGA-CESC cohorts.
